# [2-(3,5-Dimethyl-1*H*-pyrazol-1-yl-κ*N* ^2^)-1,10-phenanthroline-κ^2^ *N*,*N*′]bis­(nitrito-κ^2^ *O*,*O*′)cadmium(II)

**DOI:** 10.1107/S160053681002550X

**Published:** 2010-07-03

**Authors:** Jing Min Shi, Lin Meng, Yu Qing Wang

**Affiliations:** a Department of Chemistry, Shandong Normal University, Jinan 250014, People’s Republic of China

## Abstract

In the title complex, [Cd(NO_2_)_2_(C_17_H_14_N_4_)], the Cd^II^ ion assumes a distorted monocapped octa­hedral coordination geometry defined by an N_3_O_4_ donor set. The crystal structure is stabilized by π–π stacking inter­actions [shortest centroid–centroid distance = 3.5537 (18) Å].

## Related literature

For related structures, see: Wang *et al.* (2009 ▶); Sun *et al.* (2010 ▶).
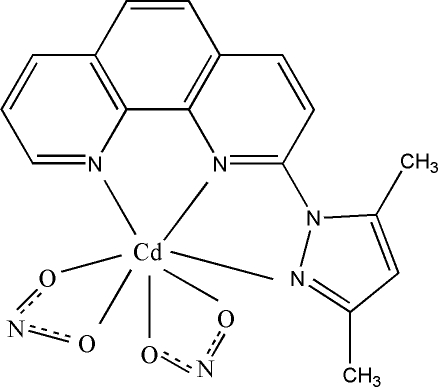


## Experimental

### 

#### Crystal data

[Cd(NO_2_)_2_(C_17_H_14_N_4_)]
*M* *_r_* = 478.74
Triclinic, 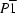

*a* = 10.0306 (15) Å
*b* = 10.4694 (15) Å
*c* = 10.5702 (15) Å
α = 67.697 (2)°
β = 83.508 (2)°
γ = 62.326 (2)°
*V* = 906.8 (2) Å^3^
*Z* = 2
Mo *K*α radiation
μ = 1.24 mm^−1^
*T* = 298 K
0.51 × 0.46 × 0.12 mm

#### Data collection

Bruker SMART APEX CCD diffractometer
Absorption correction: multi-scan (*SADABS*; Sheldrick, 1996 ▶) *T*_min_ = 0.570, *T*_max_ = 0.865
4759 measured reflections
3305 independent reflections
3098 reflections with *I* > 2σ(*I*)
*R*_int_ = 0.024

#### Refinement

*R*[*F* ^2^ > 2σ(*F* ^2^)] = 0.032
*wR*(*F* ^2^) = 0.087
*S* = 1.03
3305 reflections
255 parameters
H-atom parameters constrained
Δρ_max_ = 0.53 e Å^−3^
Δρ_min_ = −0.57 e Å^−3^

### 

Data collection: *SMART* (Bruker, 1997 ▶); cell refinement: *SAINT* (Bruker, 1997 ▶); data reduction: *SAINT*; program(s) used to solve structure: *SHELXTL* (Sheldrick, 2008 ▶); program(s) used to refine structure: *SHELXTL*; molecular graphics: *SHELXTL*; software used to prepare material for publication: *SHELXTL* and local programs.

## Supplementary Material

Crystal structure: contains datablocks I, global. DOI: 10.1107/S160053681002550X/tk2681sup1.cif

Structure factors: contains datablocks I. DOI: 10.1107/S160053681002550X/tk2681Isup2.hkl

Additional supplementary materials:  crystallographic information; 3D view; checkCIF report

## Figures and Tables

**Fig. 1.**
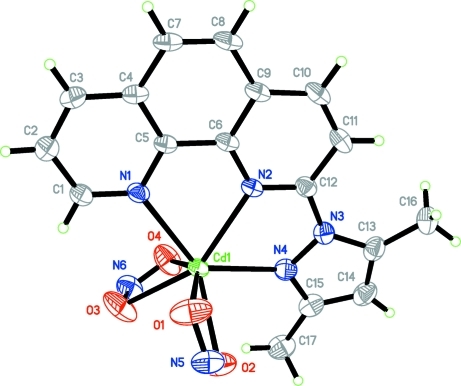
Molecular structure of (I) showing the atom-numbering scheme. Displacement ellipsoids are drawn at the 30% probability level.

## supplementary crystallographic information

### Comment

Derivatives of 1,10-phenanthroline play an important role in coordination chemistry and many complexes have been published with these molecules functioning as ligands. To our knowledge, two cadmium complexes with 2-(3,5-dimethyl-1*H*-pyrazol-1-yl)-1,10-phenanthroline derivative as the ligand have been reported (Wang *et al.*, 2009; Sun *et al.*, 2010). To study the relevance between the coordination geometry with anion, we synthesized the title complex, (I), and herein report its crystal structure.

The molecular structure of (I), Fig. 1, shows the Cd^II^ atom is coordinated by three N atoms and four O atoms within a distorted monocapped octahedral coordination geometry. The coordination geometry in (I) contrasts the penta-coordination found in the structures of the related di-chloride and di-thiocyanate derivatives (Wang *et al.*, 2009; Sun *et al.*, 2010). The non-hydrogen atoms of the 2-(3,5-dimethyl-1*H*-pyrazol-1-yl)-1,10- phenanthroline ligand define an approximate plane with a r.m.s. value = 0.0917 Å; the maximum deviation of 0.2066 (33) Å is found for the C17 atom. The crystal structure is stabilised by π–π stacking interactions with the closest of these occurring between centrosymmetrically related C4–C8 rings [*Cg*1···*Cg*1^i^ = 3.5537 (18) Å for i: 1-x, 2-y, -z].

### Experimental

A methanol (12 ml) solution of 2-(3,5-dimethyl-1*H*-pyrazol-1-yl)-1,10-phenanthroline (0.0544 g, 0.20 mmol) was added to an aqueous (12 ml) solution of CdCl_2_2.5H_2_O (0.0460 g, 0.20 mmol) and NaNO_2_ (0.0138 g, 0.20 mmol). The resultant mixture was stirred for a few minutes. The colorless swere obtained after the filtrate had been allowed to stand at room temperature for about a week.

### Refinement

All H atoms were placed in calculated positions and refined as riding with C—H = 0.96 Å and *U*_iso_ = 1.5*U*_eq_(C) for methyl-H, and C—H = 0.93 Å and *U*_iso_ = 1.2*U*_eq_(C) for the remaining H atoms.

### Crystal data

**Table:**

[Cd(NO_2_)_2_(C_17_H_14_N_4_)]	*Z* = 2
*M**_r_* = 478.74	*F*(000) = 476
Triclinic, *P*1	*D*_x_ = 1.753 Mg m^−^^3^
Hall symbol: -P 1	Mo *K*α radiation, λ = 0.71073 Å
*a* = 10.0306 (15) Å	Cell parameters from 3811 reflections
*b* = 10.4694 (15) Å	θ = 2.3–28.3°
*c* = 10.5702 (15) Å	µ = 1.24 mm^−^^1^
α = 67.697 (2)°	*T* = 298 K
β = 83.508 (2)°	Prism, colorless
γ = 62.326 (2)°	0.51 × 0.46 × 0.12 mm
*V* = 906.8 (2) Å^3^	

### Data collection

**Table:**

Bruker SMART APEX CCD diffractometer	3305 independent reflections
Radiation source: fine-focus sealed tube	3098 reflections with *I* > 2σ(*I*)
graphite	*R*_int_ = 0.024
φ and ω scans	θ_max_ = 25.5°, θ_min_ = 2.1°
Absorption correction: multi-scan (*SADABS*; Sheldrick, 1996)	*h* = −12→10
*T*_min_ = 0.570, *T*_max_ = 0.865	*k* = −12→12
4759 measured reflections	*l* = −12→8

### Refinement

**Table:**

Refinement on *F*^2^	Primary atom site location: structure-invariant direct methods
Least-squares matrix: full	Secondary atom site location: difference Fourier map
*R*[*F*^2^ > 2σ(*F*^2^)] = 0.032	Hydrogen site location: inferred from neighbouring sites
*wR*(*F*^2^) = 0.087	H-atom parameters constrained
*S* = 1.03	*w* = 1/[σ^2^(*F*_o_^2^) + (0.0563*P*)^2^ + 0.0902*P*] where *P* = (*F*_o_^2^ + 2*F*_c_^2^)/3
3305 reflections	(Δ/σ)_max_ = 0.001
255 parameters	Δρ_max_ = 0.53 e Å^−^^3^
0 restraints	Δρ_min_ = −0.57 e Å^−^^3^

### Special details

**Table:**

Geometry. All e.s.d.'s (except the e.s.d. in the dihedral angle between two l.s. planes) are estimated using the full covariance matrix. The cell e.s.d.'s are taken into account individually in the estimation of e.s.d.'s in distances, angles and torsion angles; correlations between e.s.d.'s in cell parameters are only used when they are defined by crystal symmetry. An approximate (isotropic) treatment of cell e.s.d.'s is used for estimating e.s.d.'s involving l.s. planes.
Refinement. Refinement of *F*^2^ against ALL reflections. The weighted *R*-factor *wR* and goodness of fit *S* are based on *F*^2^, conventional *R*-factors *R* are based on *F*, with *F* set to zero for negative *F*^2^. The threshold expression of *F*^2^ > σ(*F*^2^) is used only for calculating *R*-factors(gt) *etc*. and is not relevant to the choice of reflections for refinement. *R*-factors based on *F*^2^ are statistically about twice as large as those based on *F*, and *R*- factors based on ALL data will be even larger.

### Fractional atomic coordinates and isotropic or equivalent isotropic displacement parameters (Å^2^)

**Table:**

	*x*	*y*	*z*	*U*_iso_*/*U*_eq_	
C1	0.8940 (4)	0.7002 (4)	0.0995 (4)	0.0528 (8)	
H1	0.9776	0.6172	0.0866	0.063*	
C2	0.8991 (4)	0.8398 (5)	0.0647 (4)	0.0627 (9)	
H2	0.9857	0.8483	0.0314	0.075*	
C3	0.7766 (4)	0.9641 (4)	0.0796 (4)	0.0572 (8)	
H3	0.7787	1.0583	0.0559	0.069*	
C4	0.6478 (4)	0.9489 (3)	0.1308 (3)	0.0458 (7)	
C5	0.6521 (3)	0.8032 (3)	0.1659 (3)	0.0402 (6)	
C6	0.5225 (3)	0.7823 (3)	0.2193 (3)	0.0398 (6)	
C7	0.5130 (4)	1.0744 (4)	0.1465 (3)	0.0534 (8)	
H7	0.5095	1.1711	0.1224	0.064*	
C8	0.3910 (4)	1.0552 (4)	0.1956 (3)	0.0536 (8)	
H8	0.3045	1.1386	0.2046	0.064*	
C9	0.3936 (4)	0.9079 (4)	0.2340 (3)	0.0470 (7)	
C10	0.2713 (4)	0.8775 (4)	0.2884 (4)	0.0568 (8)	
H10	0.1818	0.9579	0.2976	0.068*	
C11	0.2820 (4)	0.7333 (5)	0.3275 (4)	0.0590 (9)	
H11	0.2019	0.7137	0.3652	0.071*	
C12	0.4179 (3)	0.6140 (4)	0.3094 (3)	0.0437 (7)	
C13	0.3548 (4)	0.3861 (5)	0.4020 (3)	0.0576 (9)	
C14	0.4446 (5)	0.2345 (5)	0.4242 (4)	0.0641 (10)	
H14	0.4159	0.1557	0.4593	0.077*	
C15	0.5872 (4)	0.2164 (4)	0.3855 (3)	0.0553 (8)	
C16	0.1917 (5)	0.4631 (7)	0.4248 (6)	0.0902 (15)	
H16A	0.1531	0.3888	0.4528	0.135*	
H16B	0.1371	0.5464	0.3413	0.135*	
H16C	0.1798	0.5038	0.4952	0.135*	
C17	0.7278 (5)	0.0756 (4)	0.3908 (4)	0.0706 (10)	
H17A	0.8018	0.1052	0.3416	0.106*	
H17B	0.7070	0.0186	0.3497	0.106*	
H17C	0.7658	0.0115	0.4846	0.106*	
Cd1	0.74600 (2)	0.45092 (2)	0.19946 (2)	0.04206 (11)	
N1	0.7751 (3)	0.6801 (3)	0.1503 (3)	0.0436 (6)	
N2	0.5317 (3)	0.6403 (3)	0.2551 (2)	0.0394 (5)	
N3	0.4451 (3)	0.4590 (3)	0.3502 (2)	0.0464 (6)	
N4	0.5876 (3)	0.3530 (3)	0.3395 (3)	0.0494 (6)	
N5	0.7056 (4)	0.4176 (4)	−0.0441 (4)	0.0718 (9)	
N6	1.0386 (4)	0.2533 (4)	0.3336 (4)	0.0706 (9)	
O1	0.6887 (5)	0.5379 (4)	−0.0360 (3)	0.0914 (11)	
O2	0.7445 (4)	0.3093 (4)	0.0665 (3)	0.0847 (9)	
O3	1.0138 (3)	0.3075 (5)	0.2082 (4)	0.0996 (12)	
O4	0.9256 (3)	0.2980 (4)	0.3935 (3)	0.0857 (10)	

### Atomic displacement parameters (Å^2^)

**Table:**

	*U*^11^	*U*^22^	*U*^33^	*U*^12^	*U*^13^	*U*^23^
C1	0.0421 (17)	0.0449 (18)	0.067 (2)	−0.0137 (14)	0.0081 (15)	−0.0254 (16)
C2	0.059 (2)	0.057 (2)	0.078 (2)	−0.0304 (18)	0.0145 (18)	−0.0291 (19)
C3	0.065 (2)	0.0430 (18)	0.068 (2)	−0.0264 (17)	0.0051 (17)	−0.0227 (16)
C4	0.0537 (18)	0.0334 (15)	0.0454 (16)	−0.0130 (14)	−0.0035 (13)	−0.0169 (13)
C5	0.0425 (16)	0.0332 (14)	0.0391 (15)	−0.0106 (12)	−0.0006 (12)	−0.0152 (12)
C6	0.0390 (15)	0.0357 (15)	0.0355 (14)	−0.0066 (12)	−0.0021 (11)	−0.0161 (12)
C7	0.063 (2)	0.0314 (15)	0.057 (2)	−0.0089 (15)	−0.0054 (16)	−0.0211 (14)
C8	0.0507 (19)	0.0380 (17)	0.0572 (19)	−0.0008 (14)	−0.0040 (15)	−0.0256 (15)
C9	0.0441 (17)	0.0416 (17)	0.0455 (16)	−0.0058 (13)	−0.0003 (13)	−0.0231 (14)
C10	0.0406 (17)	0.056 (2)	0.061 (2)	−0.0068 (15)	0.0097 (14)	−0.0309 (17)
C11	0.0436 (18)	0.066 (2)	0.062 (2)	−0.0187 (17)	0.0184 (15)	−0.0306 (18)
C12	0.0411 (16)	0.0480 (18)	0.0397 (15)	−0.0162 (14)	0.0030 (12)	−0.0193 (13)
C13	0.066 (2)	0.074 (3)	0.0502 (19)	−0.047 (2)	0.0113 (16)	−0.0240 (18)
C14	0.084 (3)	0.065 (2)	0.061 (2)	−0.051 (2)	0.0118 (19)	−0.0213 (18)
C15	0.070 (2)	0.0457 (19)	0.0455 (18)	−0.0277 (17)	−0.0049 (15)	−0.0077 (14)
C16	0.068 (3)	0.107 (4)	0.130 (4)	−0.058 (3)	0.038 (3)	−0.065 (3)
C17	0.082 (3)	0.041 (2)	0.075 (3)	−0.0236 (19)	−0.007 (2)	−0.0105 (17)
Cd1	0.03898 (16)	0.03134 (15)	0.04817 (16)	−0.00813 (10)	0.00277 (10)	−0.01716 (11)
N1	0.0372 (13)	0.0351 (13)	0.0507 (14)	−0.0087 (11)	0.0027 (10)	−0.0181 (11)
N2	0.0388 (13)	0.0358 (13)	0.0393 (13)	−0.0106 (10)	0.0033 (10)	−0.0179 (10)
N3	0.0468 (15)	0.0499 (16)	0.0406 (13)	−0.0229 (13)	0.0040 (11)	−0.0141 (12)
N4	0.0494 (15)	0.0377 (14)	0.0526 (15)	−0.0161 (12)	0.0000 (12)	−0.0122 (12)
N5	0.088 (2)	0.068 (2)	0.066 (2)	−0.0303 (19)	0.0007 (18)	−0.0366 (19)
N6	0.0464 (18)	0.059 (2)	0.080 (2)	−0.0031 (15)	−0.0064 (16)	−0.0233 (17)
O1	0.145 (3)	0.0523 (17)	0.0643 (18)	−0.0356 (19)	−0.0171 (19)	−0.0150 (14)
O2	0.124 (3)	0.0607 (18)	0.080 (2)	−0.0447 (19)	0.0119 (18)	−0.0346 (17)
O3	0.0577 (17)	0.108 (3)	0.087 (2)	0.0068 (17)	0.0056 (16)	−0.049 (2)
O4	0.0579 (18)	0.101 (3)	0.0636 (17)	−0.0140 (17)	0.0001 (14)	−0.0228 (16)

### Geometric parameters (Å, °)

**Table:**

C1—N1	1.325 (4)	C13—N3	1.384 (4)
C1—C2	1.390 (5)	C13—C16	1.490 (6)
C1—H1	0.9300	C14—C15	1.387 (5)
C2—C3	1.361 (5)	C14—H14	0.9300
C2—H2	0.9300	C15—N4	1.325 (4)
C3—C4	1.398 (5)	C15—C17	1.480 (5)
C3—H3	0.9300	C16—H16A	0.9600
C4—C5	1.407 (4)	C16—H16B	0.9600
C4—C7	1.430 (4)	C16—H16C	0.9600
C5—N1	1.360 (4)	C17—H17A	0.9600
C5—C6	1.435 (4)	C17—H17B	0.9600
C6—N2	1.348 (4)	C17—H17C	0.9600
C6—C9	1.398 (4)	Cd1—O1	2.339 (3)
C7—C8	1.346 (5)	Cd1—N2	2.353 (2)
C7—H7	0.9300	Cd1—N4	2.366 (3)
C8—C9	1.426 (5)	Cd1—O4	2.383 (3)
C8—H8	0.9300	Cd1—O3	2.384 (3)
C9—C10	1.414 (5)	Cd1—N1	2.405 (3)
C10—C11	1.359 (5)	Cd1—O2	2.405 (3)
C10—H10	0.9300	N3—N4	1.368 (4)
C11—C12	1.413 (4)	N5—O2	1.221 (5)
C11—H11	0.9300	N5—O1	1.226 (4)
C12—N2	1.316 (4)	N6—O4	1.215 (4)
C12—N3	1.406 (4)	N6—O3	1.231 (5)
C13—C14	1.350 (6)		
			
N1—C1—C2	122.9 (3)	C13—C16—H16C	109.5
N1—C1—H1	118.5	H16A—C16—H16C	109.5
C2—C1—H1	118.5	H16B—C16—H16C	109.5
C3—C2—C1	119.6 (3)	C15—C17—H17A	109.5
C3—C2—H2	120.2	C15—C17—H17B	109.5
C1—C2—H2	120.2	H17A—C17—H17B	109.5
C2—C3—C4	119.4 (3)	C15—C17—H17C	109.5
C2—C3—H3	120.3	H17A—C17—H17C	109.5
C4—C3—H3	120.3	H17B—C17—H17C	109.5
C3—C4—C5	117.6 (3)	O1—Cd1—N2	100.09 (11)
C3—C4—C7	123.1 (3)	O1—Cd1—N4	114.23 (12)
C5—C4—C7	119.2 (3)	N2—Cd1—N4	66.36 (9)
N1—C5—C4	122.3 (3)	O1—Cd1—O4	150.01 (13)
N1—C5—C6	118.2 (3)	N2—Cd1—O4	108.46 (10)
C4—C5—C6	119.5 (3)	N4—Cd1—O4	86.20 (11)
N2—C6—C9	122.7 (3)	O1—Cd1—O3	99.45 (13)
N2—C6—C5	118.0 (2)	N2—Cd1—O3	149.49 (12)
C9—C6—C5	119.3 (3)	N4—Cd1—O3	124.70 (12)
C8—C7—C4	121.3 (3)	O4—Cd1—O3	50.74 (11)
C8—C7—H7	119.3	O1—Cd1—N1	86.96 (10)
C4—C7—H7	119.3	N2—Cd1—N1	69.42 (8)
C7—C8—C9	120.5 (3)	N4—Cd1—N1	133.43 (8)
C7—C8—H8	119.8	O4—Cd1—N1	94.45 (11)
C9—C8—H8	119.8	O3—Cd1—N1	88.51 (12)
C6—C9—C10	115.9 (3)	O1—Cd1—O2	50.78 (11)
C6—C9—C8	120.2 (3)	N2—Cd1—O2	125.87 (11)
C10—C9—C8	124.0 (3)	N4—Cd1—O2	84.64 (10)
C11—C10—C9	121.4 (3)	O4—Cd1—O2	114.27 (13)
C11—C10—H10	119.3	O3—Cd1—O2	84.62 (13)
C9—C10—H10	119.3	N1—Cd1—O2	134.93 (10)
C10—C11—C12	118.3 (3)	C1—N1—C5	118.1 (3)
C10—C11—H11	120.9	C1—N1—Cd1	125.9 (2)
C12—C11—H11	120.9	C5—N1—Cd1	115.75 (19)
N2—C12—N3	114.7 (3)	C12—N2—C6	120.2 (2)
N2—C12—C11	121.5 (3)	C12—N2—Cd1	121.6 (2)
N3—C12—C11	123.8 (3)	C6—N2—Cd1	117.96 (19)
C14—C13—N3	105.7 (3)	N4—N3—C13	110.0 (3)
C14—C13—C16	128.3 (4)	N4—N3—C12	117.2 (2)
N3—C13—C16	126.0 (4)	C13—N3—C12	132.8 (3)
C13—C14—C15	108.2 (3)	C15—N4—N3	106.5 (3)
C13—C14—H14	125.9	C15—N4—Cd1	133.7 (2)
C15—C14—H14	125.9	N3—N4—Cd1	117.23 (19)
N4—C15—C14	109.6 (3)	O2—N5—O1	112.5 (3)
N4—C15—C17	119.7 (3)	O4—N6—O3	113.3 (3)
C14—C15—C17	130.7 (3)	N5—O1—Cd1	99.9 (2)
C13—C16—H16A	109.5	N5—O2—Cd1	96.7 (2)
C13—C16—H16B	109.5	N6—O3—Cd1	97.7 (2)
H16A—C16—H16B	109.5	N6—O4—Cd1	98.2 (2)
			
N1—C1—C2—C3	−1.6 (6)	O1—Cd1—N2—C6	−75.5 (2)
C1—C2—C3—C4	0.5 (6)	N4—Cd1—N2—C6	172.3 (2)
C2—C3—C4—C5	0.5 (5)	O4—Cd1—N2—C6	95.2 (2)
C2—C3—C4—C7	−178.4 (3)	O3—Cd1—N2—C6	53.6 (3)
C3—C4—C5—N1	−0.6 (4)	N1—Cd1—N2—C6	7.36 (19)
C7—C4—C5—N1	178.3 (3)	O2—Cd1—N2—C6	−123.9 (2)
C3—C4—C5—C6	179.8 (3)	C14—C13—N3—N4	0.8 (4)
C7—C4—C5—C6	−1.3 (4)	C16—C13—N3—N4	−178.2 (4)
N1—C5—C6—N2	2.0 (4)	C14—C13—N3—C12	−179.0 (3)
C4—C5—C6—N2	−178.4 (3)	C16—C13—N3—C12	2.0 (6)
N1—C5—C6—C9	−178.8 (3)	N2—C12—N3—N4	5.0 (4)
C4—C5—C6—C9	0.8 (4)	C11—C12—N3—N4	−173.2 (3)
C3—C4—C7—C8	179.6 (3)	N2—C12—N3—C13	−175.2 (3)
C5—C4—C7—C8	0.8 (5)	C11—C12—N3—C13	6.6 (5)
C4—C7—C8—C9	0.3 (5)	C14—C15—N4—N3	0.8 (4)
N2—C6—C9—C10	−0.4 (4)	C17—C15—N4—N3	−178.3 (3)
C5—C6—C9—C10	−179.5 (3)	C14—C15—N4—Cd1	−160.0 (2)
N2—C6—C9—C8	179.4 (3)	C17—C15—N4—Cd1	20.9 (5)
C5—C6—C9—C8	0.2 (4)	C13—N3—N4—C15	−1.0 (3)
C7—C8—C9—C6	−0.8 (5)	C12—N3—N4—C15	178.9 (2)
C7—C8—C9—C10	178.9 (3)	C13—N3—N4—Cd1	163.5 (2)
C6—C9—C10—C11	2.0 (5)	C12—N3—N4—Cd1	−16.7 (3)
C8—C9—C10—C11	−177.8 (3)	O1—Cd1—N4—C15	83.4 (3)
C9—C10—C11—C12	−1.5 (5)	N2—Cd1—N4—C15	174.1 (3)
C10—C11—C12—N2	−0.6 (5)	O4—Cd1—N4—C15	−73.9 (3)
C10—C11—C12—N3	177.6 (3)	O3—Cd1—N4—C15	−38.7 (3)
N3—C13—C14—C15	−0.3 (4)	N1—Cd1—N4—C15	−166.4 (3)
C16—C13—C14—C15	178.7 (4)	O2—Cd1—N4—C15	41.0 (3)
C13—C14—C15—N4	−0.3 (4)	O1—Cd1—N4—N3	−75.8 (2)
C13—C14—C15—C17	178.7 (4)	N2—Cd1—N4—N3	14.91 (19)
C2—C1—N1—C5	1.5 (5)	O4—Cd1—N4—N3	127.0 (2)
C2—C1—N1—Cd1	175.4 (3)	O3—Cd1—N4—N3	162.1 (2)
C4—C5—N1—C1	−0.4 (4)	N1—Cd1—N4—N3	34.4 (3)
C6—C5—N1—C1	179.2 (3)	O2—Cd1—N4—N3	−118.2 (2)
C4—C5—N1—Cd1	−174.9 (2)	O2—N5—O1—Cd1	−0.8 (4)
C6—C5—N1—Cd1	4.7 (3)	N2—Cd1—O1—N5	−128.0 (3)
O1—Cd1—N1—C1	−78.3 (3)	N4—Cd1—O1—N5	−59.5 (3)
N2—Cd1—N1—C1	179.8 (3)	O4—Cd1—O1—N5	69.8 (4)
N4—Cd1—N1—C1	160.7 (2)	O3—Cd1—O1—N5	75.5 (3)
O4—Cd1—N1—C1	71.7 (3)	N1—Cd1—O1—N5	163.5 (3)
O3—Cd1—N1—C1	21.3 (3)	O2—Cd1—O1—N5	0.5 (3)
O2—Cd1—N1—C1	−59.6 (3)	O1—N5—O2—Cd1	0.8 (4)
O1—Cd1—N1—C5	95.8 (2)	O1—Cd1—O2—N5	−0.5 (3)
N2—Cd1—N1—C5	−6.14 (19)	N2—Cd1—O2—N5	71.4 (3)
N4—Cd1—N1—C5	−25.2 (3)	N4—Cd1—O2—N5	127.0 (3)
O4—Cd1—N1—C5	−114.2 (2)	O4—Cd1—O2—N5	−149.6 (3)
O3—Cd1—N1—C5	−164.6 (2)	O3—Cd1—O2—N5	−107.3 (3)
O2—Cd1—N1—C5	114.5 (2)	N1—Cd1—O2—N5	−24.9 (3)
N3—C12—N2—C6	−176.1 (2)	O4—N6—O3—Cd1	−2.7 (4)
C11—C12—N2—C6	2.2 (4)	O1—Cd1—O3—N6	−174.7 (3)
N3—C12—N2—Cd1	9.4 (3)	N2—Cd1—O3—N6	56.1 (4)
C11—C12—N2—Cd1	−172.3 (2)	N4—Cd1—O3—N6	−46.3 (3)
C9—C6—N2—C12	−1.7 (4)	O4—Cd1—O3—N6	1.7 (3)
C5—C6—N2—C12	177.5 (3)	N1—Cd1—O3—N6	98.6 (3)
C9—C6—N2—Cd1	173.0 (2)	O2—Cd1—O3—N6	−125.9 (3)
C5—C6—N2—Cd1	−7.9 (3)	O3—N6—O4—Cd1	2.8 (4)
O1—Cd1—N2—C12	99.1 (2)	O1—Cd1—O4—N6	5.5 (4)
N4—Cd1—N2—C12	−13.1 (2)	N2—Cd1—O4—N6	−155.9 (3)
O4—Cd1—N2—C12	−90.3 (2)	N4—Cd1—O4—N6	140.6 (3)
O3—Cd1—N2—C12	−131.9 (3)	O3—Cd1—O4—N6	−1.7 (3)
N1—Cd1—N2—C12	−178.1 (2)	N1—Cd1—O4—N6	−86.1 (3)
O2—Cd1—N2—C12	50.7 (3)	O2—Cd1—O4—N6	58.2 (3)
